# A Geodetic Strain Rate Model for the East African Rift System

**DOI:** 10.1038/s41598-017-19097-w

**Published:** 2018-01-15

**Authors:** D. S. Stamps, E. Saria, C. Kreemer

**Affiliations:** 1 Virginia Tech, Department of Geosciences, 926 West Campus Drive, Blacksburg, VA 24061 USA; 2 University of California, Los Angeles, Department of Earth, Planetary, and Space Sciences, 595 Charles Young Drive East, Los Angeles, CA 90095 USA; 3 Ardhi University, Department of Geomatics, University Road, Dar Es Salaam, Tanzania; 4 University of Nevada, Nevada Bureau of Mines and Geology, 1664 N Virginia St, Reno, Nevada 89557 USA

**Keywords:** Natural hazards, Tectonics

## Abstract

Here we describe the new Sub-Saharan Africa Geodetic Strain Rate Model v.1.0 (SSA-GSRM v.1.0), which provides fundamental constraints on long-term tectonic deformation in the region and an improved seismic hazards assessment in Sub-Saharan Africa. Sub-Saharan Africa encompasses the East African Rift System, the active divergent plate boundary between the Nubian and Somalian plates, where strain is largely accommodated along the boundaries of three subplates. We develop an improved geodetic strain rate field for sub-Saharan Africa that incorporates 1) an expanded geodetic velocity field, 2) redefined regions of deforming zones guided by seismicity distribution, and 3) updated constraints on block rotations. SSA-GSRM v.1.0 spans longitudes 22° to 55.5° and latitudes −52° to 20° with 0.25° (longitude) by 0.2° (latitude) spacing. For plates/sub-plates, we assign rigid block rotations as constraints on the strain rate calculation that is determined by fitting bicubic Bessel splines to a new geodetic velocity solution for an interpolated velocity gradient tensor field. We derive strain rates, velocities, and vorticity rates from the velocity gradient tensor field. A comparison with the Global Geodetic Strain Rate model v2.1 reveals regions of previously unresolved spatial heterogeneities in geodetic strain rate distribution, which indicates zones of elevated seismic risk.

## Introduction

**Figure 1:**
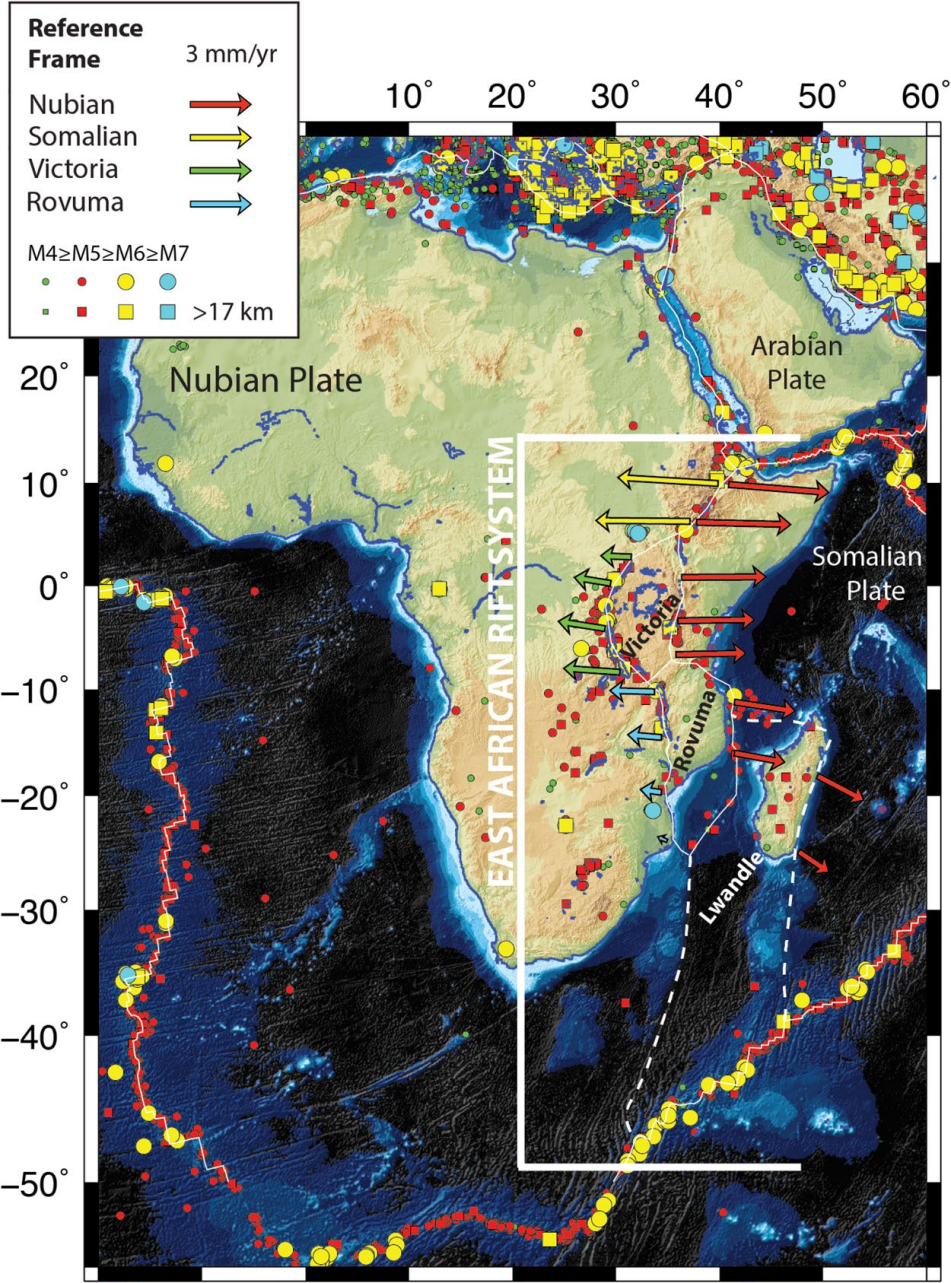
Tectonic setting of Africa and the East African Rift System. OR = Okavangu Rift, LR = Luangua Rift, MR = Mweru Rift, EB = Eastern Branch, KP = Kivu Volcanic Province, CVL = Cameroon Volcanic Line. Earthquakes >M4 from the International Seismological Catalog^65^ are shown in different colors as well as relative plate motions from Saria *et al*.^3^, which are used to constrain long-term tectonic rigid plate motions. Figure was created by DSS using the open source software Generic Mapping Tools v5.2.1 supported by the National Science Foundation.

The East African Rift System (EARS) is a seismically and volcanically active divergent plate boundary separating the Somalian and Nubian tectonic plates (Fig. 1). Hazards from seismicity along the EARS affect millions of people in the region. The Global Earthquake Model Foundation (GEM) is an international organization that aims to reduce seismic risk through developing and providing open-access products and tools for hazards reduction and risk assessment. Addressing the seismic risk in Sub-Saharan Africa (SSA) is the core of one of the GEM’s four regional projects. These regional projects are the Assessing and Mitigating Earthquake Risk in the Caribbean and Central America (CCARA) Project, South America Risk Assessment (SARA) Project, Development of an Earthquake Loss Model for Iran, and Sub-Saharan Hazard and Risk Assessment (SSAHARA) Project. In this work, we improve the GEM global geodetic strain rate model^1^ (GSRM v.2.1) for the SSA region by revising regional strain rate estimates. We resolve extensive, previously unknown/unseen spatial heterogeneities in the distribution of strain rates along the EARS.

Prior estimates of geodetic strain rates in sub-Saharan Africa from GSRM v2.1 were based largely on published geodetic data and block rotations from MORVEL^2^ in the sub-Saharan Africa region. Since the publication of GSRM v.2.1, new Global Navigation Satellite System (GNSS) observations have been obtained along the East African Rift System. In addition, Saria *et al*.^3^ published new angular velocity vectors for the Somalian and Antarctica plates, and the Victoria, Rovuma, and Lwandle subplates. Stamps *et al*.^4^ also published a continuous strain rate field for the region using both geodetic and seismic data. In this work, we incorporate only geodetic information from both the new GNSS data and Euler poles to develop SSA-GSRM v.1.0, a new strain rate model calculated using the methods of Haines and Holt^5–8^. SSA-GSRM v.1.0 provides valuable constraints on long-term tectonic deformation, which GEM can use as a basis for comparison with present-day seismic strain rates to inform hazards assessments along the EARS. SSA-GSRM v1.0 and the velocity solution can also be used for tectonic investigations of the region.

## Methods

### Mesh Geometry

**Figure 2:**
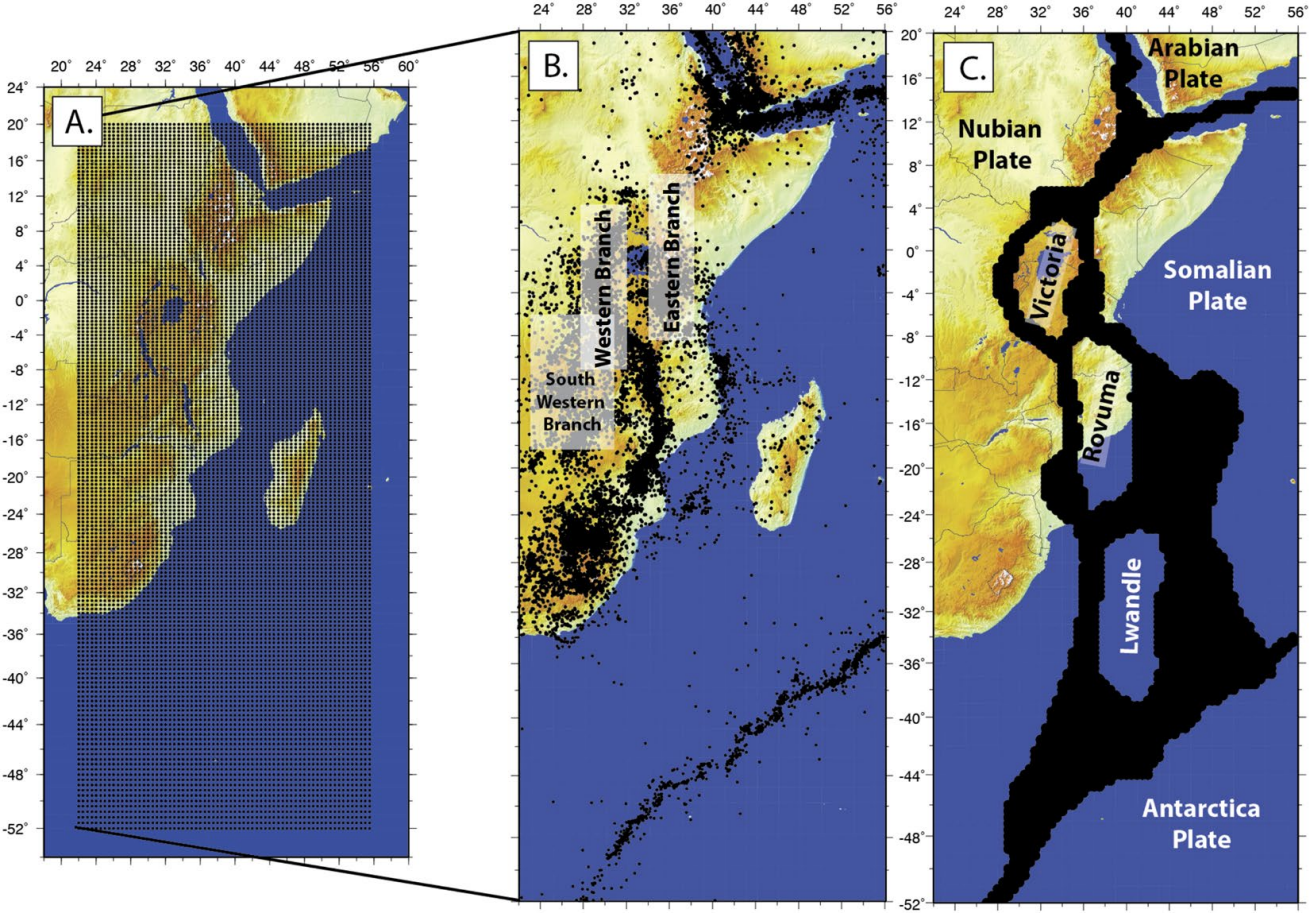
Depiction of the mesh geometry and its development into deforming zones. (**A**) Mesh geometry. (**B**) Seismicity from the International Seismological ISC catalog showing earthquakes ≥M2, which was used to guide the location of deforming zones depicted in black in (**C**). All figures were created by DSS using the open source software Generic Mapping Tools v5.2.1 supported by the National Science Foundation.

Our mesh for the sub-Saharan Africa region encompasses longitudes 26° to 55.5° and latitude −52° to 20° with a grid spacing of 0.25° in longitude and 0.2° in latitude (Fig. 2A). We select these values for grid spacing because they are compatible with GSRM v.2.1 The chosen size of the grid cells is smaller than the typical station spacing, and we do not have the data to constrain such high-resolution model. While we do not have the data in many places to resolve the strain rate field at the resolution of the grid cells, the Haines and Holt approach ensures that strain rates will spatially vary slowly there. The small grid cell size is only chosen to allow for integration of SSA-GSRM v.1.0 into the next version of GSRM. We define regions of deformation by first using seismicity from the International Seismicity Catalog (Fig. 2B), the locations of existing GNSS observations, and previous studies that indicate rigidity^9–11^. Along the EARS, we outline zones of profuse active seismicity and then reduce the spatial extent of these zones since there are currently no GNSS observations available to constrain the possible deformation, or the region is rigid within observational error^10,11^. In particular, we note seismicity in the Southwestern Branch of the EARS associated with active deformation in the Okavangu, Mweru, Luangua rifts, the Cameroon volcanic line region, southwest Botswana, and regions in South Africa. However, we do not model these active zones as deforming because of the reasons described above.

### Geodetic Velocity Solution

The new GNSS data used in this work to constrain deformation in sub-Saharan Africa are a combination of both continuous and episodic GNSS observations (See Supplementary dataset 1). We require at least two and a half years of observations for continuous GNSS data to minimize seasonal signals in the time-series^12^. Episodic GNSS sites must have at least three or more occupations of at least forty-eight hours, each spanning four or more years. We removed GNSS data from the velocity solution that have observed transient movement due to magmatic activity. GNSS stations in the Dabahhu dike region in the northern EARS and the Natron region in the southern Eastern Branch adjacent to the Gelai dike are identified based on previous studies^13,14^. The Kivu Rift region is also identified as having magmatic activities^15^, which is monitored by the KivuGNet GNSS network^16^. In this region, a separate analysis by Ji *et al*.^17^ of seven continuous time-series isolates the tectonic signal from volcanic deformation signals by applying principal component analysis to the time-series. In the Kivu Rift region, we use the corrected tectonic GNSS signal as a constraint for the long-term deformation.

**Figure 3:**
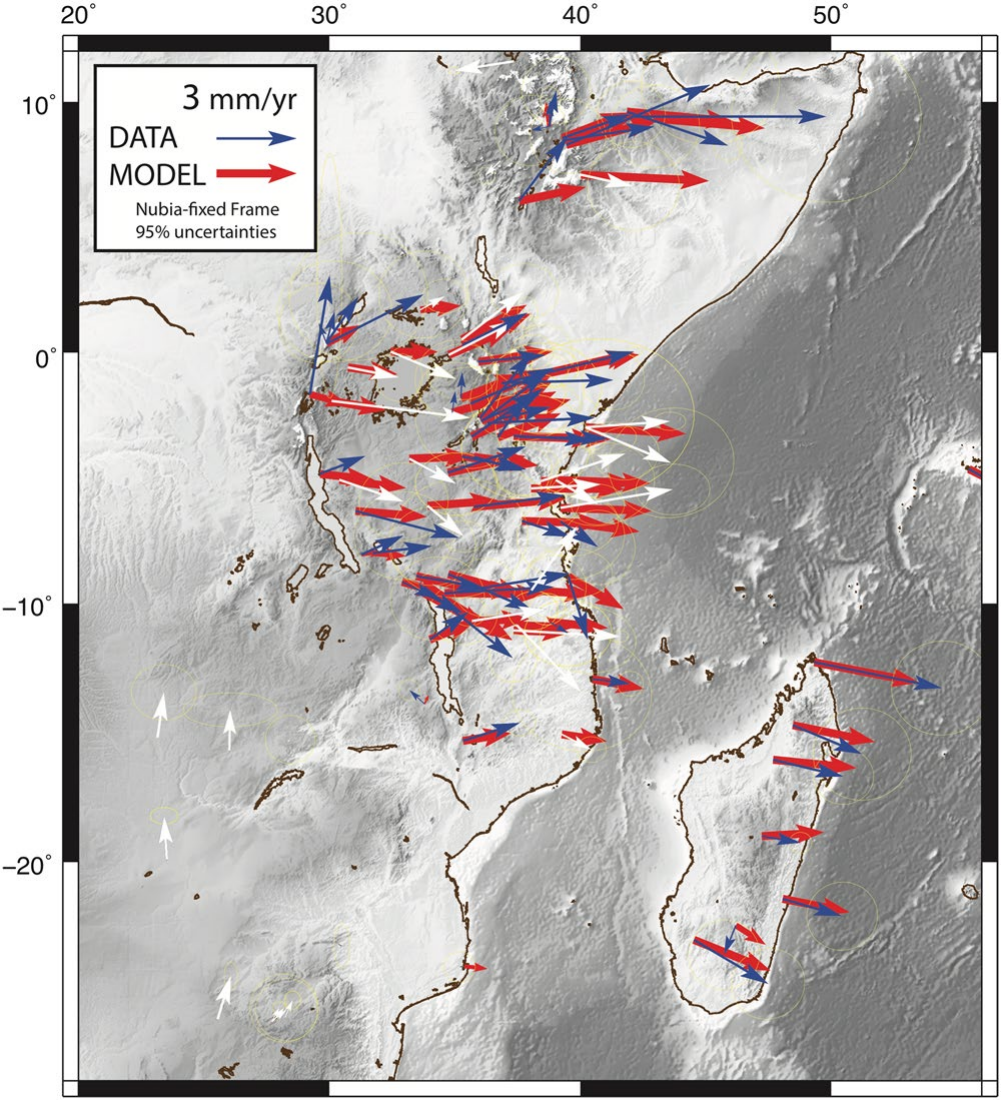
GNSS velocity solution shown as blue and white vectors with 95% uncertainty ellipses and the modeled velocities from SSA-GSRM v1.0 are shown in red. Red and blue vectors are in deforming zones and white vectors are not used because they are in rigid zones constrained by the Saria *et al*.^3^ angular velocity vectors. All figures were created by DSS using the open source software Generic Mapping Tools v5.2.1 supported by the National Science Foundation.

We calculate a geodetic solution comprised of publicly accessible data (Solution A) and a separate solution (Solution B) that includes newly acquired episodic GNSS data in Tanzania^18^, Uganda^19^, and Madagascar^20^ (Fig. 3). Additional data shown in Figure 3 include^21–58^. The full set of data references are provided in a column of the supplementary velocity solution velocity.csv file. The two solutions are combined using common sites ABPO, TANZ, EBBE, MBAR, SRTI, REUN, and SEY1 by calculating a fourteen parameter Helmert transformation of position and velocity. For a Nubia-fixed reference frame, the velocity solutions of fifty-six common sites are used. The consistency of the sites in the Nubian reference frame is obtained with an RMS value of 0.68 mm/yr for the fifty-six common sites. The two GNSS solutions (A and B) are processed using a three-step approach with GAMIT-GLOBK processing software^59^. For the processing strategies, readers are referred to^3,60,61^.

### Geodetic Strain Rate Calculation

We use the methods of Haines and Holt^5–8^ to calculate SSA-GRSM v.1.0 (See Supplementary dataset 2), which is a continuous strain rate field constrained by geodetic observations. SSA-GSRM v.1.0 can be used as a constraint on long-term tectonic deformation to inform present-day hazards assessment along the EARS. We use this approach for compatibility with the GSRM v.2.1^1^. The Haines and Holt method involves defining zones of rigidity and deformation a priori and fitting bicubic Bessel splines to an interpolation of irregularly spaced velocities across pre-defined deforming zones at plate boundaries. To account for sparse GNSS station distribution and to fit deforming regions with relatively higher strain rates compared to the rigid zones, this method allows for the application of spatially-varying a priori (co)variances to each mesh element in the deforming regions. We follow the method of Kreemer *et al*.^1^ for the GEM geodetic strain rate model, which involves a two-step approach. First, we assign uniform variances with standard deviations 10^−9^ yr^−1^ and 1/√2 * 10^−9^ yr^−1^ for the diagonal ${\varepsilon ^{\prime} }_{xx,yy}$, and off-diagonal ${\varepsilon ^{\prime} }_{xy}$ components of the strain rate tensor, respectively. In this first step, the region is considered isotropic; thus, we assign zero covariances. In the second step, we use the second invariant:

1 $$\,\varOmega =\sqrt{{{\varepsilon ^{\prime} }_{xx}}^{2}+{{\varepsilon ^{\prime} }_{yy}}^{2}+2{{\varepsilon ^{\prime} }_{xy}}^{2}}$$

and $\varOmega /\sqrt{2}$ of the strain rate calculation from the first step to constrain the a priori standard deviations for the diagonal and off-diagonal components of the strain rate tensor, respectively. If we instead decided to apply uniform variances and co-variances in the second step, we would have unrealistic strain across the plate boundary zones where there is minimal or no GNSS data. For example, if we did not follow the two-step approach of Kreemer *et al*.^1^, then deformation across Madagascar and the Rovuma plate boundary would not be localized to Madagascar near the available GNSS observations. Rather, deformation would be smoothed across the entire region with minimal lateral variations in strain that are detectable with GNSS data.

### Data availability

Geodesy Data Facility UNAVCO (www.unavco.org, doi:10.7283/T5SN077J, doi:10.7283/T5WS8RKK, doi:10.7283/T5XD0ZZG). Solution B is comprised of the velocity solution from Saria *et al*.^3^. The final combined solution (Fig. 3, blue vectors) for this work can be found in the supplemental data file velocity.csv with all available data references.

## Results

**Figure 4:**
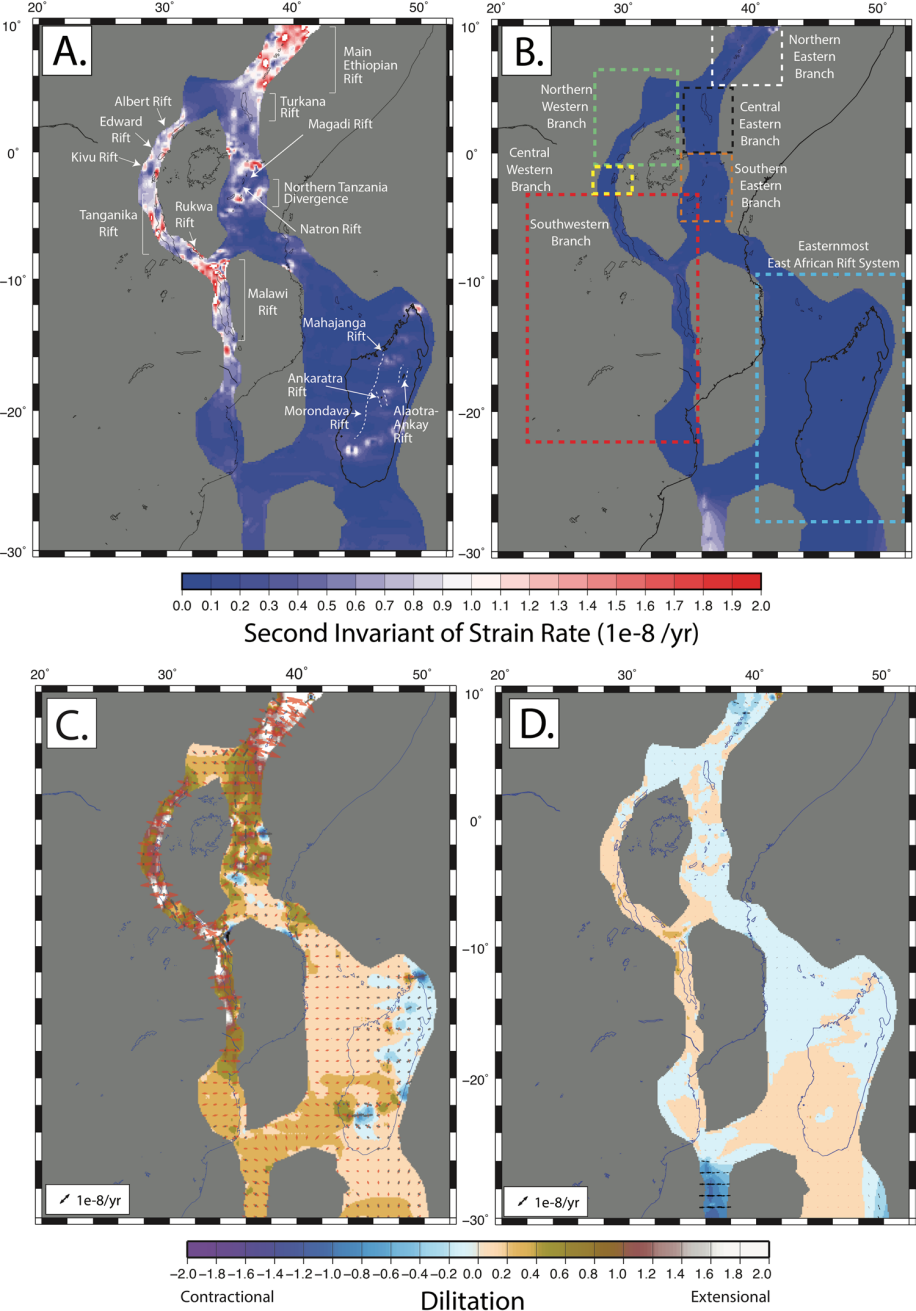
Geodetic strain rate second invariant and dilatation and comparison with GSRM v.2.1. (**A**) The second invariant of strain rate for the new long-term tectonic deformation model indicating magnitude. (**B**) Residual strain rate magnitudes relative to GSRM v2.1. (**C**) Dilatation indicating the dominantly compressional and extensional regimes. Tensor orientations are overlaid. Red = extension and black = compression. (**C**,**D**) Same as (**C**), but for residual strain rate tensors and dilatation. All figures were created by DSS using the open source software Generic Mapping Tools v5.2.1. supported by the National Science Foundation.

The new SSA-GSRM v.1.0 derived from GNSS velocities and angular velocity vector constraints in rigid regions fit the GNSS observations with a weighted root mean square of 1.99 mm/yr (Fig. 3). Strain rate magnitudes (Ω) in the SSA range from ~0–2 * 10^−8^ yr^−1^ with the highest strain rates localized in the Main Ethiopian Rift, the Tanganika Rift, and the intersection of the Victoria-Nubia-Rovuma plates near the Rukwa Rift and northern Malawi Rift (Fig. 4A). Relatively higher geodetic strain rates are also evident along the northern and central Western Branches in the Albert Rift, Edward Rift, and Kivu Rift. In the central and southern Eastern Branch, we find the Turkana Rift has relatively lower geodetic strain rates than the Magadi Rift, Natron Rift, and Northern Tanzanian Divergence to the south, which may be due to a sparsity of observations in the region. Our geodetic strain rate magnitudes are within two nanostrains of the global strain rate model (Fig. 4B). Figure 4C shows the trace, or dilatation:

2 $$({\varepsilon ^{\prime} }_{xx}+{\varepsilon ^{\prime} }_{yy})$$

of the geodetic strain tensor indicative of dominantly compressional or tensional strain. In our model, strain is largely E-W extensional across the Eastern and Western Branches with larger magnitudes of strain rates within deforming zones and low strain rates in rigid plate interiors, as expected per our definition of rigid and deforming zones. Figure 4D is a comparison of the dilatation with the global geodetic strain rate model. We find the largest differences in the northern Eastern Branch along the Main Ethiopian Rift, the Tanganika Rift, and along the Malawi Rift. Each of the aforementioned locations suggests our study finds more extension in these regions. We also find a minor amount of compression in northern and south-central Madagascar near the Mahajanga, Morondava, Alaotra-Ankay, and Ankaratra Rifts. We resolve these differences because of new GNSS observations across the island^20,57,58^. Overall, geodetic strain rates along the EARS are characterized by low magnitude extensional deformation with small, spatially varying regions of compression along branches of the central EARS, and widespread low magnitude transpression in the easternmost EARS.

## Discussion

**Figure 5:**
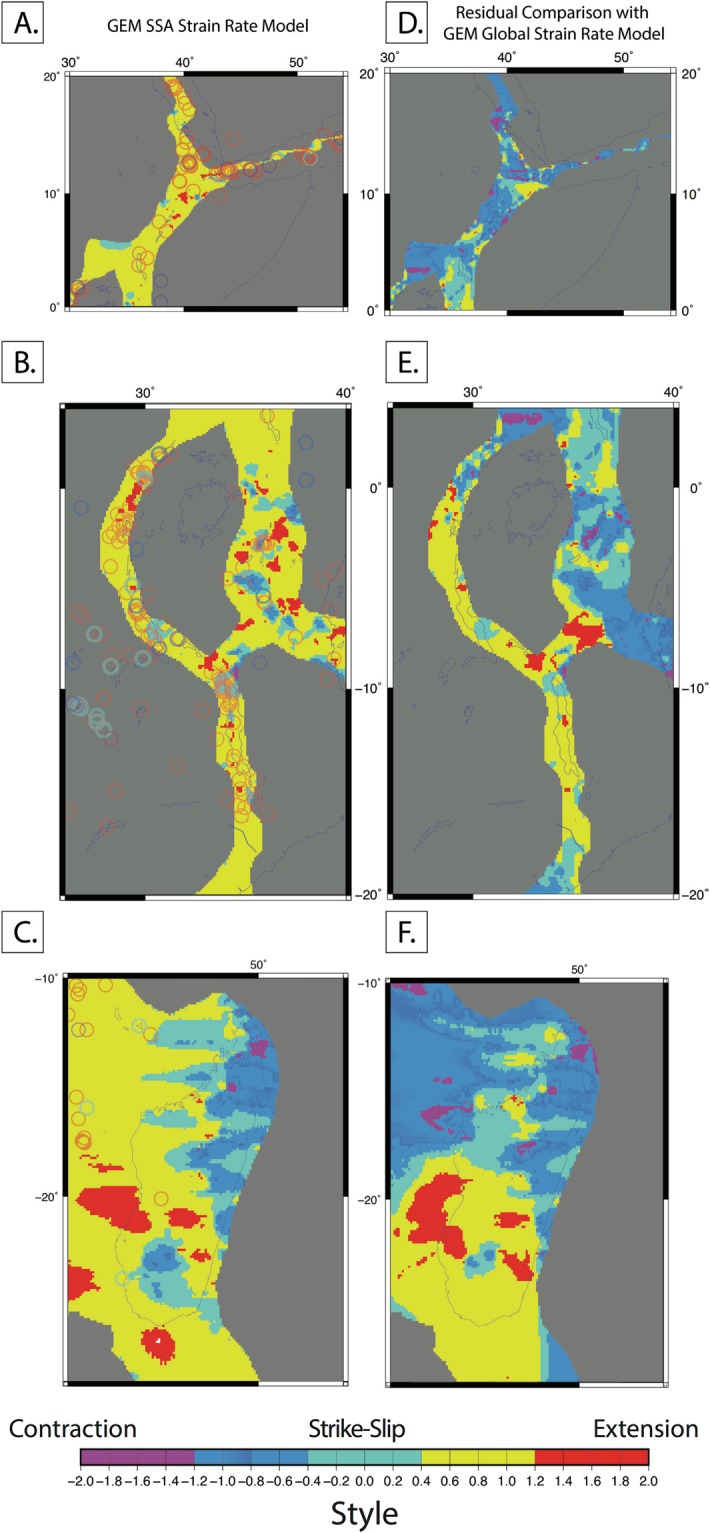
SSA-GSRM v1.0 strain styles and comparison with GRSM v2.1. (**A**–**C**) are the predicted tectonic strain styles for the northern EARS, central EARS, and easternmost EARS respectively. Moment tensor styles are overlain as transparent colored circles with the same scale as the background style map as documented in the World Stress Map Database^27^. The color scale for the background tectonic style map is determined by equally dividing the rake values. (**D**–**F**) Residuals compared to global GEM geodetic strain rate model styles. All figures were created by DSS using the open source software Generic Mapping Tools v5.2.1.

Here, we compare the geodetic strain rate styles of the new SSA-GSRM v.1.0 to GSRM v.2.1 (Fig. 5), which we define as:

3 $$({\varepsilon ^{\prime} }_{1}+{\varepsilon ^{\prime} }_{2})/{\rm{\max }}({|\varepsilon ^{\prime} |}_{1},|{\varepsilon ^{\prime} }_{2}|)$$

(Kreemer *et al*.^1^) where ${\varepsilon ^{\prime} }_{1}\,\text{and}\,{\varepsilon ^{\prime} }_{2}$ are, respectively, the largest and smallest eigenvectors. Overall, SSA-GSRM v.1.0 resolves more spatial variability of tectonic strain styles due to the increase in GNSS observations when compared with GSRM v.2.1. In the northern Eastern Branch along the Main Ethiopian Rift, we find two GNSS-constrained zones of transpression amongst tensional strain that deforms slower than the GEM global model because we used different Somalian plate angular rotations (Fig. 5A,D). In the northern and central Western Branch, we resolve zones of strike-slip and transtensional deformation in the Albert, Edward, and Kivu Rifts where GSRM v.2.1 exhibits only extension (Fig. 5B,E). In the Southwestern Branch both models are similar with transtension across the Rukwa Rift and unresolvable strain elsewhere. In the Eastern Branch, SSA-GSRM v1.0 indicates tectonic deformation is expected in a variety of styles rather than dominantly extensional as in the GEM global strain rate model. A zone of transpression is expected in southern Madagascar according to GSRM v.2.1. However, we find minor strain accumulation across the continental island in the form of transtension and transpression. These comparisons suggest the EARS is accommodating extension more heterogeneously than previously expected.

The EARS remains one of the least monitored tectonic plate boundaries, which makes it challenging to constrain present-day seismic hazards. Our long-term tectonic strain rate model provides a foundation for comparison with present-day seismic data^62^. It can be used for elucidating where strain may be accumulating towards better informing hazards assessment and reducing risk. In Fig. 5A–C, we overlay the style of present-day deformation determined from focal mechanisms along the EARS, as documented in the World Stress Map^63^. We find extensional events, that are either purely normal or with a dip-slip component, and that are consistent with most of the expected deformation due to tectonics. Seismic event styles are fully consistent with our model in the northern EARS (Fig. 5A), however, in the central EARS the fit varies. Notable positive correlations are in the northern Western Branch and south of the Northern Tanzania Divergence, where compressional or thrust-strike-slip events are expected. A clear mismatch occurs in the northern Malawi Rift (Fig. 5B), where geodetic strains are expected as pure strike-slip movements, but earthquakes are purely normal faulting events. The easternmost EARS in Madagascar shows one compression-strike-slip event that is near a region in southern Madagascar, where tectonic deformation is consistent^63^ (Fig. 5C). There are mostly extensional and extension-dominated events that correlate with expected tectonic deformation west of Madagascar. However, three compressional events are not indicated in our tectonic model – possibly due to the sparsity of our observations in the region. Recent earthquakes have occurred outside of the zones, in which we define as deforming, i.e. the April 2017 Mw 6.5 Moiyabana, Botswana, earthquake^64^. This discrepancy highlights a limitation of this technique in that we can only calculate strain rates in regions where we have observations. This comparison of our expected tectonic deformation patterns and present-day seismicity highlights the need for continued investigations into the influence of long-term tectonic deformation on present-day hazards, including the need for additional instrumentation in zones of active seismicity.

### Final Remarks

Our new geodetic strain rate field is a foundational tectonic strain rate model for the EARS based on new GNSS observations and an open access contribution to the Global Earthquake Model Foundation (GEM) strain rate project for the sub-Saharan Africa (SSA-GSRM v.1.0). We provide gridded strain rate values, vorticity, and directions of no-length-change. We have used methods that are complementary to GSRM v.2.1 such that future iterations of GSRM can integrate this work. We compare SSA-GSRM v1.0 with the Global Strain Rate Model v2.1 (GSRM v2.1) and find more spatial variations in strain rate style across the Eastern and Western Branches, the Main Ethiopian Rift, and Madagascar that were not resolved in GSRM v2.1. This work can be used to improve our ability to assess earthquake potential in sub-Saharan Africa by providing a baseline strain rate model that is representative of long-term tectonic deformation for comparison with present-day seismic strain rates.

## Electronic supplementary material

Supplementary Information

Dataset 1

Dataset 2
