# Author Correction: A Geodetic Strain Rate Model for the East African Rift System

**DOI:** 10.1038/s41598-019-56280-7

**Published:** 2020-01-08

**Authors:** D. S. Stamps, E. Saria, C. Kreemer

**Affiliations:** 1 Virginia Tech, Department of Geosciences, 926 West Campus Drive, Blacksburg, VA 24061 USA; 2 University of California, Los Angeles, Department of Earth, Planetary, and Space Sciences, 595 Charles Young Drive East, Los Angeles, CA 90095 USA; 3 Ardhi University, Department of Geomatics, University Road, Dar Es Salaam, Tanzania; 4 University of Nevada, Nevada Bureau of Mines and Geology, 1664 N Virginia St, Reno, Nevada 89557 USA

Correction to: *Scientific Reports* 10.1038/s41598-017-19097-w, published online 15 January 2018

The original version of this Article contained extensive errors in the Reference list where several data references were omitted. The omitted references are now listed as references 21–56 and the original references 21–23 are listed now as references 59–61, the original references 24–25 are listed now as references 57–58 and the original references 26–29 are now listed as references 62–65.

As such, the legend of Figure 1,

“Tectonic setting of Africa and the East African Rift System. OR = Okavangu Rift, LR = Luangua Rift, MR = Mweru Rift, EB = Eastern Branch, KP = Kivu Volcanic Province, CVL = Cameroon Volcanic Line. Earthquakes > M4 from the International Seismological Catalog^29^ are shown in different colors as well as relative plate motions from Saria *et al*.^3^, which are used to constrain long-term tectonic rigid plate motions. Figure was created by DSS using the open source software Generic Mapping Tools v5.2.1 supported by the National Science Foundation.”

now reads:

“Tectonic setting of Africa and the East African Rift System. OR = Okavangu Rift, LR = Luangua Rift, MR = Mweru Rift, EB = Eastern Branch, KP = Kivu Volcanic Province, CVL = Cameroon Volcanic Line. Earthquakes > M4 from the International Seismological Catalog^65^ are shown in different colors as well as relative plate motions from Saria *et al*.^3^, which are used to constrain long-term tectonic rigid plate motions. Figure was created by DSS using the open source software Generic Mapping Tools v5.2.1 supported by the National Science Foundation.”

The Methods section under subheading ‘Geodetic Velocity Solution’,

“We calculate a geodetic solution comprised of publicly accessible data (Solution A) and a separate solution (Solution B) that includes newly acquired episodic GNSS data in Tanzania^18^, Uganda^19^, and Madagascar^20^ (Fig. 3). The two solutions are combined using common sites ABPO, TANZ, EBBE, MBAR, SRTI, REUN, and SEY1 by calculating a fourteen parameter Helmert transformation of position and velocity. For a Nubia-fixed reference frame, the velocity solutions of fifty-six common sites are used. The consistency of the sites in the Nubian reference frame is obtained with an RMS value of 0.68 mm/yr for the fifty-six common sites. The two GNSS solutions (A and B) are processed using a three-step approach with GAMIT-GLOBK processing software^21^. For the processing strategies, readers are referred to^3,22,23^.”

now reads:

“We calculate a geodetic solution comprised of publicly accessible data (Solution A) and a separate solution (Solution B) that includes newly acquired episodic GNSS data in Tanzania^18^, Uganda^19^, and Madagascar^20^ (Fig. 3). Additional data shown in Figure 3 include^21-58^. The full set of data references are provided in a column of the supplementary velocity solution velocity.csv file. The two solutions are combined using common sites ABPO, TANZ, EBBE, MBAR, SRTI, REUN, and SEY1 by calculating a fourteen parameter Helmert transformation of position and velocity. For a Nubia-fixed reference frame, the velocity solutions of fifty-six common sites are used. The consistency of the sites in the Nubian reference frame is obtained with an RMS value of 0.68 mm/yr for the fifty-six common sites. The two GNSS solutions (A and B) are processed using a three-step approach with GAMIT-GLOBK processing software^59^. For the processing strategies, readers are referred to^3, 60,61^.”

The data availability statement,

“Geodesy Data Facility UNAVCO (www.unavco.org, doi:10.7283/T5SN077J, doi:10.7283/T5WS8RKK, doi:10.7283/T5XD0ZZG). Solution B is comprised of the velocity solution from Saria *et al*.^3^. The final combined solution (Fig. 3, blue vectors) for this work can be found in the supplemental data file velocity.csv.”

now reads:

“Geodesy Data Facility UNAVCO (www.unavco.org, 10.7283/T5SN077J, 10.7283/T5WS8RKK, 10.7283/T5XD0ZZG). Solution B is comprised of the velocity solution from Saria *et al*.^3^. The final combined solution (Fig. 3, blue vectors) for this work can be found in the supplemental data file velocity.csv with all available data references.”

The results section,

“We resolve these differences because of new GNSS observations across the island^20,24,25^.”

now reads:

“We resolve these differences because of new GNSS observations across the island^20,57,58^.”

The discussion section,

“The EARS remains one of the least monitored tectonic plate boundaries, which makes it challenging to constrain present-day seismic hazards. Our long-term tectonic strain rate model provides a foundation for comparison with present-day seismic data^26^. It can be used for elucidating where strain may be accumulating towards better informing hazards assessment and reducing risk. In Fig. 5A–C, we overlay the style of present-day deformation determined from focal mechanisms along the EARS, as documented in the World Stress Map^27^. We find extensional events, that are either purely normal or with a dip-slip component, and that are consistent with most of the expected deformation due to tectonics. Seismic event styles are fully consistent with our model in the northern EARS (Fig. 5A), however, in the central EARS the fit varies. Notable positive correlations are in the northern Western Branch and south of the Northern Tanzania Divergence, where compressional or thrust-strike-slip events are expected. A clear mismatch occurs in the northern Malawi Rift (Fig. 5B), where geodetic strains are expected as pure strike-slip movements, but earthquakes are purely normal faulting events. The easternmost EARS in Madagascar shows one compression-strike-slip event that is near a region in southern Madagascar, where tectonic deformation is consistent^27^ (Fig. 5C). There are mostly extensional and extension-dominated events that correlate with expected tectonic deformation west of Madagascar. However, three compressional events are not indicated in our tectonic model – possibly due to the sparsity of our observations in the region. Recent earthquakes have occurred outside of the zones, in which we define as deforming, i.e. the April 2017 Mw 6.5 Moiyabana, Botswana, earthquake^28^.”

now reads:

“The EARS remains one of the least monitored tectonic plate boundaries, which makes it challenging to constrain present-day seismic hazards. Our long-term tectonic strain rate model provides a foundation for comparison with present-day seismic data^62^. It can be used for elucidating where strain may be accumulating towards better informing hazards assessment and reducing risk. In Fig. 5A–C, we overlay the style of present-day deformation determined from focal mechanisms along the EARS, as documented in the World Stress Map^63^. We find extensional events, that are either purely normal or with a dip-slip component, and that are consistent with most of the expected deformation due to tectonics. Seismic event styles are fully consistent with our model in the northern EARS (Fig. 5A), however, in the central EARS the fit varies. Notable positive correlations are in the northern Western Branch and south of the Northern Tanzania Divergence, where compressional or thrust-strike-slip events are expected. A clear mismatch occurs in the northern Malawi Rift (Fig. 5B), where geodetic strains are expected as pure strike-slip movements, but earthquakes are purely normal faulting events. The easternmost EARS in Madagascar shows one compression-strike-slip event that is near a region in southern Madagascar, where tectonic deformation is consistent^63^ (Fig. 5C). There are mostly extensional and extension-dominated events that correlate with expected tectonic deformation west of Madagascar. However, three compressional events are not indicated in our tectonic model – possibly due to the sparsity of our observations in the region. Recent earthquakes have occurred outside of the zones, in which we define as deforming, i.e. the April 2017 Mw 6.5 Moiyabana, Botswana, earthquake^64^.”

Additionally, the Acknowledgements section,

“Materials in this work are based on data and equipment services provided by the UNAVCO Facility with support from the National Science Foundation (NSF) and National Aeronautics and Space Administration (NASA) under NSF Cooperative Agreement No. EAR-0735156. We are grateful for the efforts of the International GNSS Service for providing data products and access to publicly accessible GNSS data. GNSS data collection by D.S. Stamps was funded by USAID and NSF EAR Postdoctoral Fellowship Program #EAR-1249295.”

now reads:

“Materials in this work are based on data and equipment services provided by the UNAVCO Facility with support from the National Science Foundation (NSF) and National Aeronautics and Space Administration (NASA) under NSF Cooperative Agreement No. EAR-0735156. We are grateful for the efforts of the International GNSS Service for providing data products and access to publicly accessible GNSS data. GNSS data collection by D.S. Stamps was funded by USAID, NSF EAR Postdoctoral Fellowship Program #EAR-1249295, National Geographic Society/Waitt Grant Program # W193-11, and National Geographic Society Grant # 9501-14.”

Finally, in the Supplementary Information file originally published with this Article, a column with data DOI’s and associated paper citations was omitted.

These errors have been corrected in the HTML and PDF versions of the original Article, alongside the Supplementary Information that accompanies the Article.

