# Supplementary material for: A Geodetic Strain Rate Model for the East African Rift System
Source: Sci Rep. 2018 Jan 15;8:732. doi: 10.1038/s41598-017-19097-w (PMC5768676; doi:10.1038/s41598-017-19097-w)
Supplement: Supplementary file 1 — Supplementary Information [file 41598_2017_19097_MOESM1_ESM.pdf]

A Geodetic Strain Rate Model for the East African Rift System

D. S. Stamps[1,2], E. Saria[3], C. Kreemer[4]

[1] Virginia Tech, Department of Geosciences, 925 West Campus Drive, Blacksburg, VA, 24061, dstamps@vt.edu

[2] University of California, Los Angeles, Department of Earth, Planetary, and Space Sciences, 595 Charles Young Drive East, Los Angeles, CA 90095, dstamps@ucla.edu

[3] Ardhi University, Department of Geomatics, University Road, Dar Es Salaam, Tanzania

[4] University of Nevada, Nevada Bureau of Mines and Geology, 1664 N Virginia St, Reno, Nevada, 89557

SUPPLEMENTARY MATERIAL

velocity.csv

The combined velocity solution used in this study to calculate the Sub-Saharan Africa Geodetic Strain Rate Model 1.0 in comma separated format. The columns are longitude, latitude, velocity East, velocity North, sigma East, sigma North, correlation.

model.txt.zip

The Sub-Saharan Africa Geodetic Strain Rate Model 1.0 formatted as latitude, longitude, strain rate components xx, yy, xy, vorticity, no length change right-lateral, no length change left lateral, principal strain rates e1 and e2, and the azimuth of the principal strain rate e1.
